# Phosphorylation of Serine 235 of the Hepatitis C Virus Non-Structural Protein NS5A by Multiple Kinases

**DOI:** 10.1371/journal.pone.0166763

**Published:** 2016-11-22

**Authors:** Kuan-Ying Lee, Yi-Hung Chen, Shih-Chin Hsu, Ming-Jiun Yu

**Affiliations:** Institute of Biochemistry and Molecular Biology, National Taiwan University College of Medicine, Taipei, 10051, Taiwan; Taipei Medical University, TAIWAN

## Abstract

Phosphorylation at serine 235 (S235) of the hepatitis C virus (HCV) non-structural protein 5A (NS5A) plays a critical role in the viral life cycle. For medical and virological interests, we exploited the HEK293T kidney cells to test 3 candidate protein kinases on NS5A S235 phosphorylation. Inhibitors that inhibit casein kinase I α (CKIα), polo-like kinase I (PlKI) or calmodulin-dependent kinase II (CaMKII) all reduced NS5A S235 phosphorylation. CKIα was studied previously and PlKI had severe cytotoxicity, thus CaMKII was selected for validation in the Huh7.5.1 liver cells. In the HCV (J6/JFH1)-infected Huh7.5.1 cells, CaMKII inhibitor reduced NS5A S235 phosphorylation and HCV RNA levels without apparent cytotoxicity. RT-PCR analysis showed expression of CaMKII γ and δ isoforms in the Huh7.5.1 cells. Both CaMKII γ and δ directly phosphorylated NS5A S235 in vitro. CaMKII γ or δ single knockdown did not affect NS5A S235 phosphorylation but elevated the HCV RNA levels in the infected cells. CKIα plus CaMKII (γ or δ) double knockdown reduced NS5A S235 phosphorylation and reduced HCV RNA levels; however, the HCV RNA levels were higher than those in the infected cells with CKIα single knockdown. We conclude that CKIα-mediated NS5A S235 phosphorylation is critical for HCV replication. CaMKII γ and δ may have negative roles in the HCV life cycle.

## Introduction

Hepatitis C virus (HCV) is an enveloped virus with a positive single-stranded RNA genome. The viral genome encodes a polyprotein that is processed by the host and viral proteases into 3 structural (core, E1 and E2) and 7 non-structural (p7, NS2, NS3, NS4A, NS4B, NS5A and NS5B) proteins [1]. The structural proteins together with the host membranes make up the viral particles whereas the non-structural proteins are essential for a complete HCV life cycle. Many approved high efficiency drugs target the non-structural proteins for HCV infection that often leads to fibrosis, cirrhosis and cancer, if left unattended [2]. For example, there are drugs that target the non-structural proteins with apparent enzymatic activities i.e. the NS3/4A protease complex and the RNA-dependent RNA polymerase NS5B [1, 3, 4]. There are also drugs targeting NS5A that does not have apparent enzymatic functions [5–9]. How these NS5A drugs work is not entirely understood.

NS5A is a multi-functional protein participating in HCV replication and assembly [10, 11]. NS5A’s functions are regulated in part by its phosphorylation states i.e. hypo- and hyper-phosphorylation that appear as protein bands at 56 and 58 kDa on immunoblot. A series of serine residues in the low complexity sequence region I (LCS-I) of NS5A is responsible for NS5A hyper-phosphorylation and functions [12–15]. For example, alanine mutations in serine 225, 229, 232 and 235 in the LCS-I region reduce NS5A hyper-phosphorylation and reduce genotype 2 HCV replication [12, 14, 15]. NS5A hyper-phosphorylation at S225 and S232 also participates in viral assembly [15]. Regardless the exact functions of NS5A hyper-phosphorylation, efforts have been made to develop drugs that inhibit NS5A hyper-phosphorylation [16]. The approved NS5A drug daclatasvir, for example, inhibits NS5A hyper-phosphorylation and membranous web formation required for viral replication [8, 9]. Daclatasvir was also shown to bind NS5A dimer and interrupt its RNA binding ability thereby reducing viral replication [5–7].

Previously using a phosphorylation-specific antibody, we showed that S235 of NS5A is phosphorylated in the HCV (J6/JFH1 genotype 2a)-infected Huh7.5.1 cells [12]. The S235 phosphorylated NS5A corresponds to the hyper-phosphorylated NS5A and its phosphorylation levels correlates with the viral replication activity. Casein kinase I α (CKIα) directly phosphorylates NS5A S235 in vitro [12]. Reducing CKIα activity with an inhibitor or small RNA-mediated knockdown reduces S235 phosphorylation and viral replication. Chemicals and alanine mutation that affect NS5A phosphorylation at S235 reduced viral replication [12, 14, 15, 17]. These observations prompted us to devise a proof-of-principle platform for screening kinases involved in NS5A S235 phosphorylation using the transfection-friendly HEK293T cells that were shown to support the HCV life cycle when expressing the liver-specific micro-RNA 122 [18, 19]. Using this system, we identified calmodulin-dependent kinase II (CaMKII) that can directly phosphorylate NS5A at S235 in vitro. However, CKIα is likely the major kinase responsible for NS5A hyper-phosphorylation and viral replication in vivo.

## Results

### The HEK293T kidney cells recapitulated NS5A phosphorylation as in the HCV-infected Huh7.5.1 liver cells

**Fig 1:**
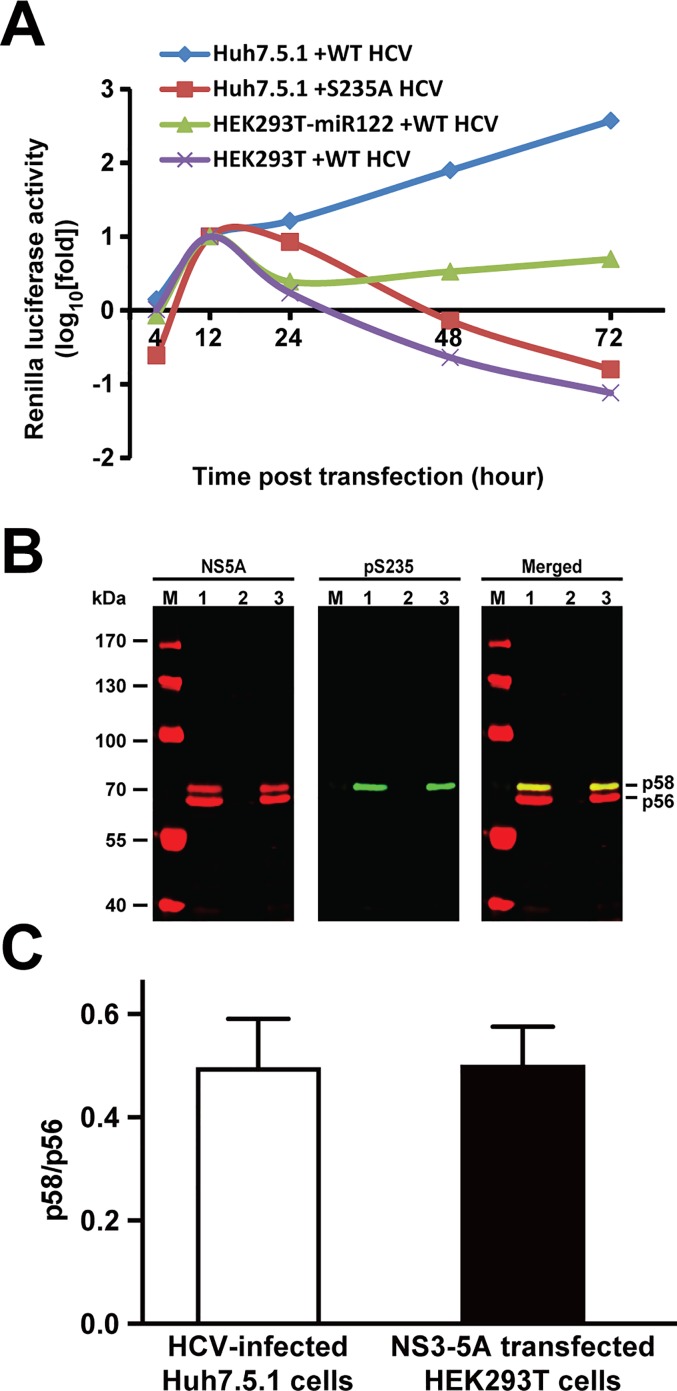
The HEK293T kidney cells recapitulated HCV NS5A phosphorylation and functions as seen in the HCV-infected Huh7.5.1 liver cells. (A) Reporter virus assay. The HEK293T cells were transfected with the wild type (WT) full length reporter HCV genomic RNA (5’C19Rluc2AUbi) with or without the miR-122 expression vector before the measurements of the Renilla luciferase activity. The Huh7.5.1 cells transfected with the full length WT or S235A replication-defective reporter HCV genomic RNA serves as controls. (B) Representative and (C) summary of the immunoblotting for NS5A and NS5A phosphorylation at serine 235 (pS235) in the HCV (J6/JFH1)-infected Huh7.5.1 cells (7 days after infection, lane 1) and in the HEK293T cells transfected without (lane 2) or with the NS3-5A expression construct (2 days after transfection, lane 3). Lane M indicates protein size markers. Hypo- and hyper-phosphorylated NS5A are labeled as p56 and p58, respectively. Protein abundance was measured with the Li-Cor Odyssey scanner and software. Values are Mean ± SEM (n = 3).

Before using the HEK293T cells as a kinase screening platform for HCV NS5A phosphorylation, we first tested the cells for their ability to support the HCV life cycle. The HEK293T cells were transfected with the Renilla reporter HCV RNA with or without the liver-specific miR-122 before the reporter activity was measured. As has been shown previously [18], the non-hepatic HEK293T cells were able to support the reporter HCV activity when the liver-specific miR-122 was co-expressed (Fig 1A, green line), albeit at a lower level compared to those in the Huh7.5.1 liver host cells (blue line). The HEK293T cells without expressing miR-122 could not sustain the reporter activity (Fig 1A, purple line) that kept decreasing with time similar to that observed for the replication-defective S235A mutant (red line) [12]. Thus, the HEK293T cells contain essential protein components required for the HCV life cycle. In the HCV-infected Huh7.5.1 cells, NS5A appeared as two characteristic bands at 56 and 58 kDa known as hypo- and hyper-phosphorylated NS5A on the immunoblot (Fig 1B, left panel, lane 1). NS5A phosphorylation at S235 was apparent and corresponded to the hyper-phosphorylated band in the HCV-infected cells (Fig 1B, middle and right panels, lane 1). In the HEK293T cells transfected with the NS3-5A expression construct (without miR-122 expression), NS5A also appeared as the two characteristic phosphorylated NS5A bands (Fig 1B, left panel, lane 3). NS5A phosphorylation at S235 was also apparent and corresponded to the hyper-phosphorylated band in the NS3-5A expressing HEK293T cells (Fig 1B, middle and right panels, lane 3). The ratios of p58 over p56 in the NS3-5A expressing HEK293T cells were similar to those in the HCV-infected Huh7.5.1 cells (Fig 1C). Therefore, the HEK293T cells host similar protein kinase and phosphatase repertoire for NS5A phosphorylation and hence a reasonable model for screening kinases and phosphatases involved in NS5A phosphorylation. The HEK293T cells expressing NS3-5A without miR-122 expression were used for screening NS5A kinases in order to avoid complication of the HCV life cycle that would alter the NS5A protein levels and interpretation of the drug effects on NS5A phosphorylation vs. viral life cycle.

### Calmodulin and calmodulin-dependent kinase inhibitors reduced NS5A S235 phosphorylation in the HEK293T cells

**Fig 2:**
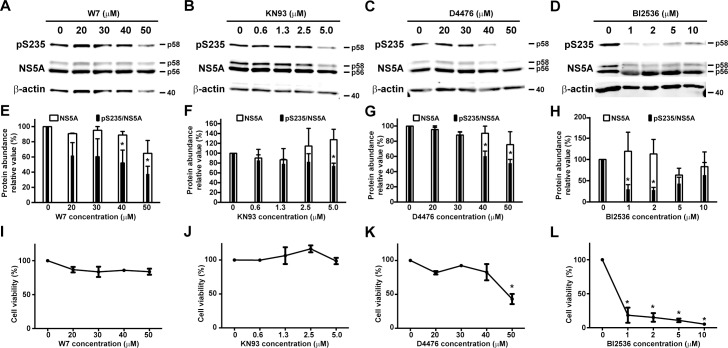
Calmodulin, CaMKII, CKIα and PlkI inhibitors reduced HCV NS5A S235 phosphorylation in the HEK293T cells. (A-D) Representative and (E-H) summary of the immunoblotting for NS5A and NS5A phosphorylation at S235 (pS235) in the NS3-5A transfected HEK293T cells. One day after the transfection, the cells were exposed to the inhibitors for 2 days before the immunoblotting analysis. Values are Mean ± SEM (n = 3). Asterisk indicates significance i.e. p<0.05, t-test against the values in the vehicle controls (0 μM). Protein intensities were measured with the Li-Cor Odyssey scanner and software and adjusted for the loadings with the β-actin intensity. Relative abundance was normalized against the values under the vehicle control (0 μM). (I-L) Cell viability assessed with the MTT assay. The cells were exposed to the inhibitors for 2 days before the viability assay. Values are Mean ± SEM (n = 3).

As a proof of principle, we elected three protein kinases to test their functions in NS5A S235 phosphorylation: casein kinase I α (CKIα) [20–22] and polo-like kinase I (PlkI) [23] already known to phosphorylate NS5A plus calmodulin-dependent kinase II (CaMKII) predicted to phosphorylate NS5A S235 in our kinase motif analysis [24]. The NS3-5A expressing HEK293T cells were exposed to inhibitors for calmodulin (W7), CKIα (D4476), PlkI (BI2536) and CaMKII (KN93) before immunoblotting for NS5A and NS5A S235 phosphorylation and cell viability assay. All inhibitors in a dose-dependent manner reduced the ratios of NS5A S235 phosphorylation (pS235/NS5A) in the NS3-5A expressing HEK293T cells while the total NS5A stayed relatively unaffected (Fig 2). W7, KN93, D4476 and BI2536 significantly reduced the ratios of S235 phosphorylation at 40, 5, 40 and 1 μM, respectively. All inhibitors, except the PlkI inhibitor, did not affect cell viability at the above effective doses.

### Calmodulin and calmodulin-dependent kinase inhibitors reduced NS5A S235 phosphorylation and HCV RNA levels in the infected Huh7.5.1 cells

**Fig 3:**
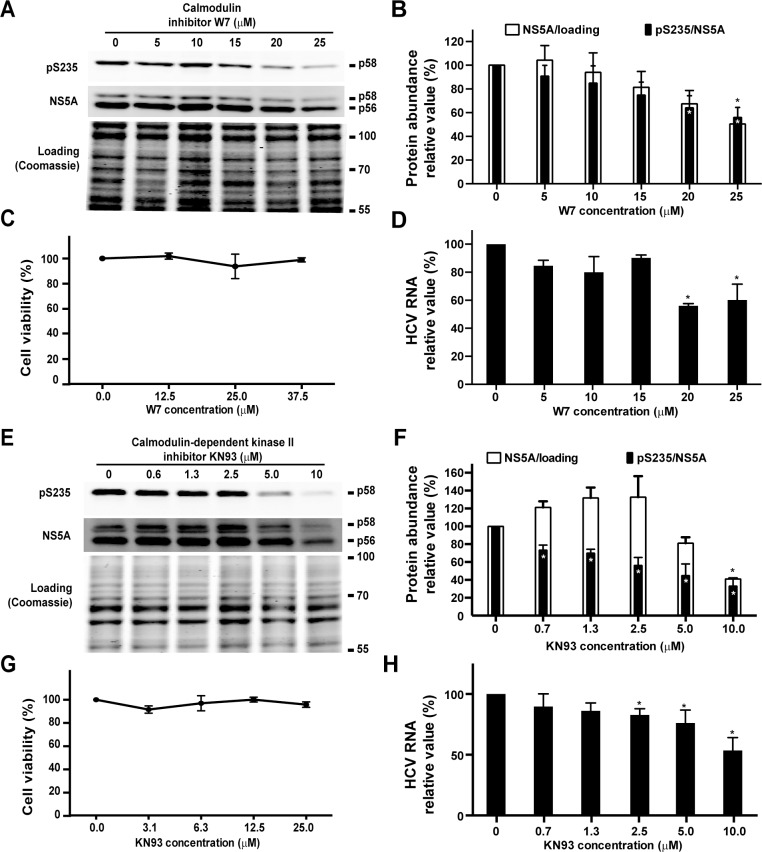
Calmodulin and CaMKII inhibitors reduced NS5A S235 phosphorylation and HCV RNA levels in the infected Huh7.5.1 cells. (A and E) Representative and (B and F) summary of the immunoblotting for NS5A and NS5A phosphorylation at S235 (pS235) in the HCV (J6/JFH1)-infected Huh7.5.1 cells. The HCV-infected cells (3 days) were exposed to the inhibitors for 1 day before the immunoblotting analysis. Values are Mean ± SEM (n = 3). Asterisk indicates significance i.e. p<0.05, t-test against the values in the vehicle controls (0 μM). Protein abundance was quantified with the Li‐Cor scanner, adjusted for the loadings (i.e. Coomassie staining) and normalized with the values in the vehicle control cells (0 μM). (C and G) Cell viability assessed with the MTT assay. The cells were exposed to the inhibitors for 1 day before the MTT assay. (D and H) Quantitative measurements of the HCV RNA levels in the HCV-infected Huh7.5.1 cells exposed to the inhibitors. The experiments were done as those in a-c and e-g except that RNA was collected for the analysis. The GAPDH mRNA levels were analyzed in parallel for the loadings. Relative RNA abundance was normalized to the values of the vehicle controls (0 μM).

Because CKIα was studied previously [22] and the PlkI inhibitor had severe cytotoxicity (Fig 2I), CaMKII was studied further in the HCV (J6/JFH1)-infected Huh7.5.1 cells. As shown in the immunoblotting results, both calmodulin inhibitor W7 (Fig 3A and 3B) and CaMKII inhibitor KN93 (Fig 3E and 3F) significantly reduced the ratios of NS5A S235 phosphorylation in the HCV-infected cells in a dose dependent manner. The reduction in the NS5A S235 phosphorylation ratios was accompanied with significant reduction in the total NS5A protein levels (Fig 3A, 3B, 3E and 3F) and in the HCV RNA levels (Fig 3D and 3H), consistent with a reduction in the HCV replication by the inhibitors. The effective doses of W7 (20 μM) and KN93 (2.5 μM) did not exert cytotoxicity in the Huh7.5.1 cells (Fig 3C and 3G).

### Calmodulin-dependent kinase II γ and δ are expressed in the Huh7.5.1 cells

**Fig 4:**
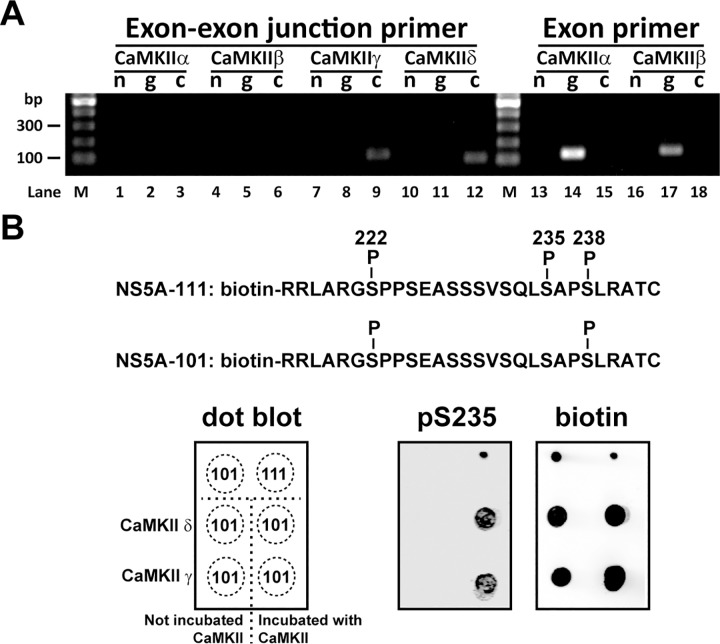
CaMKII γ and δ expressed in the Huh7.5.1 cells can directly phosphorylate NS5A S235. (A) Expression of CaMKII isoforms in the Huh7.5.1 cells by RT-PCR analysis. Expression of CaMKII (γ and δ) mRNA was assessed with the genomic (g) and cDNA (c) libraries of the Huh7.5.1 cells using primers that span two neighboring exons (exon-exon junction primer) to avoid false positive signal from the genomic DNA. No template (n) served as negative controls. Lack of CaMKII (α and β) mRNA expression was double checked with primer pairs within the same exons (exon primer). The lanes are numbered. Lane M indicates DNA markers in base pairs (bp). (B) In vitro kinase assay. Three digits were used to denote the phosphorylation status of the three serine residues 222, 235 and 238 in the biotin-labeled synthetic NS5A peptide (NS5A-111 and NS5A-101). Numbers 1 and 0 represent phosphorylation and non-phosphorylation, respectively.

There are 4 CaMKII isoforms (α, β, γ and δ) in human [25]. We found that only CaMKII γ and δ mRNA was expressed in the Huh7.5.1 cells based on RT-PCR analysis of the cDNA library (Fig 4A, lanes 9 and 12) using primers that span two neighboring exons that avoid false amplification from the genomic DNA (lanes 8 and 11). No CaMKII α or β mRNA was detected in the cDNA library of the Huh7.5.1 cells using primers that span two exons (Fig 4A, lanes 3 and 6) or primers that are within the same exons (lanes 15 and 18). As positive controls for the RT-PCR, the latter primer pairs did produce clear PCR products from the genomic DNA (Fig 4A, lanes 14 and 17).

### Calmodulin-dependent kinase II γ and δ directly phosphorylated NS5A at S235

To test whether CaMKII γ or δ directly phosphorylates NS5A at S235, two NH_2_-terminally biotin-labeled NS5A peptides were synthesized (Fig 4B). The NS5A-111 peptide was synthesized with S222, S235 and S238 phosphorylated whereas the NS5A-101 peptide was synthesized with S222 and S238 phosphorylated. S222 and S238 phosphorylation were included in the peptides to account for the fact that S222 and S238 phosphorylation were identified in our previous protein mass spectrometry [12]. As seen in the dot blot result (Fig 4B, pS235), the S235 phosphorylation-specific antibody detected the NS5A-111 but not the NS5A-101 peptide, indicating that the antibody is specific to S235 phosphorylation and not S222 or S238 phosphorylation. Peptide loadings were controlled by the biotin blot (Fig 4B, biotin). The dot blot result also indicates that the detection of S235 phosphorylation by the antibody was not interfered with when both S222 and S238 are phosphorylated on the same peptide. When the NS5A-101 peptide was incubated with the CaMKII γ or δ kinase, S235 phosphorylation was detected (Fig 4B, pS235). S235 phosphorylation was not detected when the NS5A-101 peptide was not incubated with the kinases. These results indicate that CaMKII γ or δ can directly phosphorylate NS5A S235 in vitro.

### Calmodulin-dependent kinase II γ or δ knockdown did not affect NS5A S235 phosphorylation in the infected Huh7.5.1 cells

**Fig 5:**
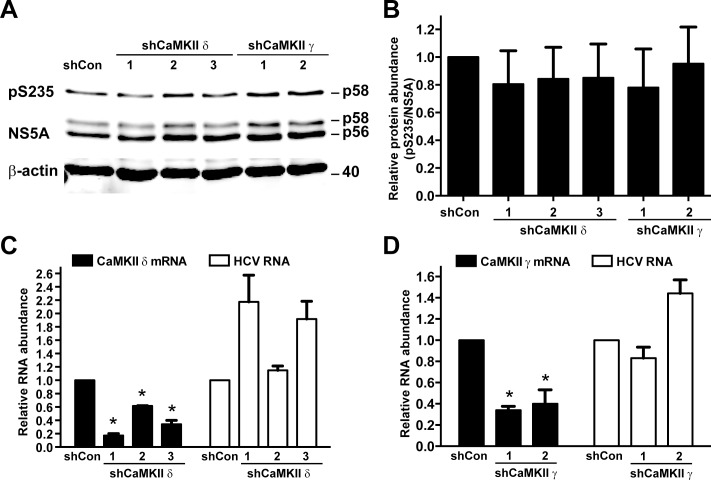
CaMKII γ or δ knockdown did not affect NS5A S235 phosphorylation or HCV RNA levels in the infected Huh7.5.1 cells. (A) Representative and summary (B) of the immunoblotting for NS5A and NS5A phosphorylation at S235 (pS235) in the HCV-infected Huh7.5.1 cells upon CaMKII γ or δ knockdown. One day after the cells were infected with HCV (J6/JFH1), the cells were subjected to small hairpin RNA (shRNA)-mediated CaMKII knockdown for 6 days prior to the immunoblotting analysis. Three and two shRNA sequences were used for CaMKII δ and γ Knockdown. Values are Mean ± SEM (n = 3). Asterisk indicates significance i.e. p<0.05, t-test against the values in the non-targeted control cells (shCon). Protein abundance was quantified with the Li‐Cor scanner, adjusted for the loadings (i.e. against β-actin) and normalized with the values in the control cells. (C and D) CaMKII mRNA and HCV RNA levels measured with quantitative RT-PCR in the HCV-infected Huh7.5.1 cells with or without CaMKII knockdown. The experiments were done as those in a and b except that RNA was collected for the analysis. The GAPDH mRNA levels were analyzed as loading controls. Relative RNA abundance was normalized to the values of the control cells.

To test whether CaMKII γ or δ is responsible for NS5A S235 phosphorylation in vivo, CaMKII γ or δ was knocked down with 2 or 3 shRNA sequences targeting different regions of the CaMKII mRNA in the HCV (J6/JFH1)-infected Huh7.5.1 cells followed by immunoblotting for NS5A S235 phosphorylation. Fig 5A shows the representative immunoblotting results. Fig 5B summarizes results from 3 independent experiments. As seen, CaMKII γ or δ knockdown did not affect the ratios of NS5A phosphorylation at S235 over total NS5A, indicating that CaMKII γ or δ may not be the kinase responsible for NS5A S235 phosphorylation in vivo. In fact, CaMKII γ or δ knockdown appeared to elevate the HCV RNA levels (Fig 5C and 5D, white bars), suggesting that CaMKII γ or δ could potentially suppress the HCV life cycle. The CaMKII knockdown efficiency was verified with quantitative RT-PCR measurements of the CaMKII γ or δ mRNA levels (Fig 5C and 5D, black bars). There were not CaMKII isoform-specific antibodies available to double check the knockdown efficiency at the protein levels.

### CKIα plus CaMKII γ or δ double knockdown reduced NS5A S235 phosphorylation and HCV RNA levels in the infected Huh7.5.1 cells

**Fig 6:**
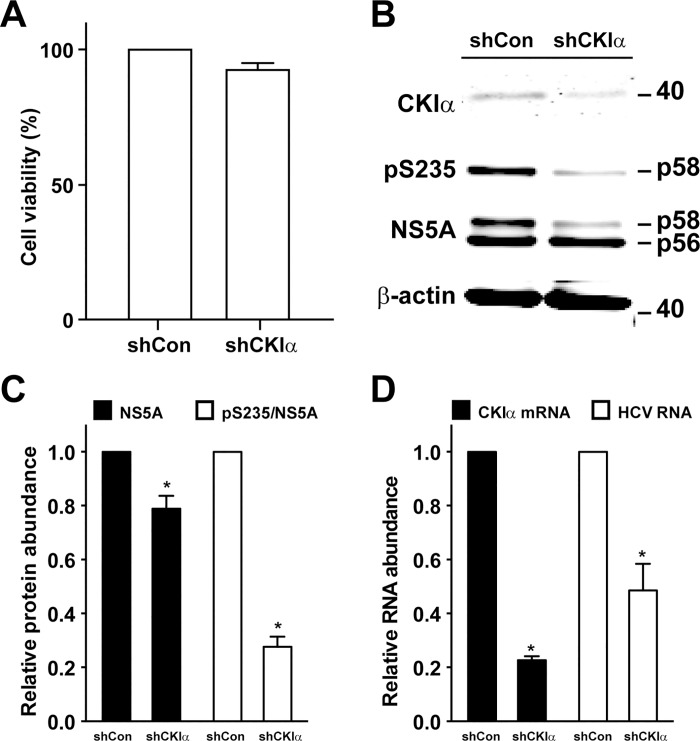
CKIα knockdown reduced NS5A S235 phosphorylation and HCV RNA levels in the infected Huh7.5.1 cells. (A) Viability of the Huh7.5.1 cells with or without CKIα knockdown assessed with the MTT assay. (B) Representative and summary (C) of the immunoblotting for CKIα, NS5A and NS5A phosphorylation at S235 (pS235) in the HCV-infected Huh7.5.1 cells with or without CKIα knockdown. One day after the cells were infected with HCV (J6/JFH1), the cells were subjected to shRNA-based CKIα knockdown for 6 days prior to the immunoblotting analysis. Values are Mean ± SEM (n = 3). Asterisk indicates significance i.e. p<0.05, t-test against the values in the non-targeted control cells (shCon). Protein abundance was quantified with the Li‐Cor scanner, adjusted for the loadings (i.e. against β-actin) and normalized with the values in the control cells. (D) CKIα mRNA and HCV RNA levels measured with quantitative RT-PCR in the HCV-infected Huh7.5.1 cells with or without CKIα knockdown. The experiments were done as those in b except that RNA was collected for the analysis. The GAPDH mRNA levels were analyzed as loading controls. Relative RNA abundance was normalized with the values of the control cells.

Given CKIα’s role in NS5A S235 phosphorylation [12], we tested whether the lack of effects of CaMKII γ or δ knockdown was due to compensatory activity of CKIα. To this end, we performed single CKIα knockdown or double CKIα plus CaMKII δ or γ knockdown in the HCV (J6/JFH1)-infected Huh7.5.1 cells followed by immunoblotting for NS5A S35 phosphorylation. CKIα single knockdown did not affect the viability of the Huh7.5.1 cells (Fig 6A). Fig 6B shows the representative immunoblotting results. Fig 6C summarizes results from 3 independent experiments. CKIα single knockdown significantly reduced the ratios of NS5A S235 phosphorylation overall total NS5A to about 28% compared to the ratios in the control cells (Fig 6C, white bars). The reduction in the ratios of NS5A S235 phosphorylation paralleled with reduction in the total NS5A protein levels to about 79% (Fig 6C, black bars) and the HCV RNA levels to about 49% (Fig 6D, white bars) in the HCV-infected Huh7.5.1 cells, consistent with an inhibition in HCV replication upon CKIα knockdown. The CKIα knockdown efficiency was verified at the mRNA levels (Fig 6D, black bars) and at the protein levels (Fig 6B).

**Fig 7:**
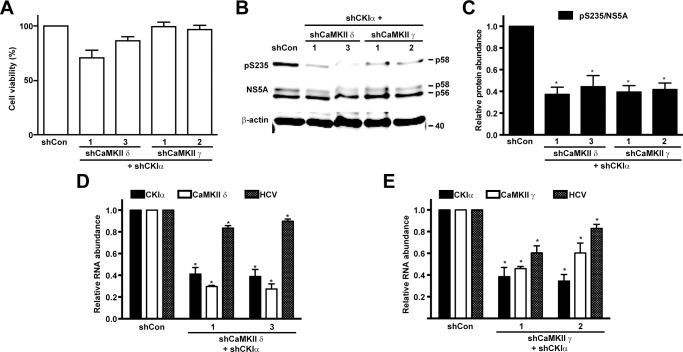
CKIα plus CaMKII double knockdown reduced NS5A S235 phosphorylation and HCV RNA levels in the infected Huh7.5.1 cells. (A) Viability of the Huh7.5.1 cells with CKIα plus CaMKII double knockdown. (B) Representative and summary (C) of the immunoblotting for NS5A and NS5A phosphorylation at S235 (pS235) in the HCV-infected Huh7.5.1 cells with or without CKIα plus CaMKII double knockdown. One day after the cells were infected with HCV (J6/JFH1), the cells were subjected to shRNA-based double knockdown for 6 days prior to the immunoblotting analysis. Values are Mean ± SEM (n = 3). Asterisk indicates significance i.e. p<0.05, t-test against the values in the non-targeted control cells (shCon). Protein abundance was quantified with the Li‐Cor scanner, adjusted for the loadings (i.e. against β-actin) and normalized with the values in the control cells. (D and E) Quantitative measurements of kinase mRNA and HCV RNA in the HCV-infected Huh7.5.1 cells with or without CKIα plus CaMKII double knockdown. The experiments were done as those in b except that RNA was collected for the analysis. The GAPDH mRNA levels were analyzed as loading controls. Relative RNA abundance was normalized to the values of the control cells.

CKIα plus CaMKII (γ or δ) double knockdown did not affect the viability of the Huh7.5.1 cells (Fig 7A). Compared to CKIα single knockdown (Fig 6C, white bars), CKIα plus CaMKII (γ or δ) double knockdown did not further reduce the ratios of NS5A S235 phosphorylation (Fig 7B and 7C). The HCV RNA levels in the CKIα plus CaMKII (γ or δ) double knockdown cells were lower than those in the control cells (Fig 7D and 7E, hashed bars); however, they were higher than those in the CKIα single knockdown cells (Fig 6D, white bars). The efficiency of the double knockdown was verified at the mRNA levels (Fig 7D and 7E, black and white bars). These data are consistent with a predominant role of CKIα-mediated NS5A S235 phosphorylation in HCV replication in vivo [12] and suggest a negative role of CaMKII γ or δ in the HCV life cycle.

## Discussion

In this study, we exploited the NS3-5A expressing HEK293T kidney cells as a screening platform for kinases involved in NS5A S235 phosphorylation, a critical post-translational modification required for HCV replication [12, 14, 15]. The HEK293T cells were selected over the HCV host liver cells for their high transfection efficiency and for their ability to support the reporter HCV activity when the liver specific miR-122 was expressed in the kidney cells [18]. As shown (Fig 1A), the miR-122 expressing HEK293T cells were able to support the HCV reporter virus activity, indicating that the HEK293T cells contain necessary protein components for the HCV life cycle. In fact, when the HEK293T cells were transfected with the NS3-5A expression construct, NS5A appeared as two characteristic hypo- and hyper-phosphorylated bands with a ratio very similar to that seen in the HCV-infected Huh7.5.1 liver host cells (Fig 1B and 1C). Therefore, the HEK293T cells contain kinases and phosphatases for NS5A phosphorylation and hence a reasonable model for screening NS5A kinases. As positive controls, we tested two kinases, CKIα [12, 20, 21] and PlkI [23], previously reported to be responsible for NS5A hyper-phosphorylation plus a candidate kinase, CaMKII, that was predicted to phosphorylate S235 by a bioinformatic program [24]. The CaMKII inhibitor, like the inhibitors for the positive control kinases, reduced NS5A hyper-phosphorylation and S235 phosphorylation in a dose-dependent manner in the NS3-5A expressing HEK293T cells (Fig 2), suggesting that CaMKII is involved in NS5A S235 phosphorylation. The above results were verified in the HCV host liver cells Huh7.5.1 (Fig 3E–3H). We further showed that CaMKII γ and δ were expressed in the Huh7.5.1 cells (Fig 4A) and that both CaMKII γ and δ can directly phosphorylate NS5A at S235 in vitro (Fig 4B). Thus, the NS3-5A expressing HEK293T cells, as a proof of principle, can be exploited to screen for kinases involved in site-specific NS5A phosphorylation. Another advantage is that the HEK293T cells do not support HCV life cycle without miR-122 expression. This avoids complication due to HCV propagation that affects NS5A protein levels. If the effects of the kinase inhibitors were examined in the miR-122 expressing HEK293T cells or HCV-infected Huh7.5.1 cells i.e. in the context of the HCV life cycle, one could not distinguish whether the kinase inhibitors could affect NS5A S235 phosphorylation alone or as a result of secondary effects on the HCV life cycle.

Although the HEK293T cell-based screening suggested a role of CaMKII in NS5A S235 phosphorylation, the results that we obtained from the Huh7.5.1 cells using chemical-mediated CaMKII inhibition and shRNA-mediated CaMKII knockdown were quite different. While the KN93-mediated CaMKII inhibition reduced NS5A S235 phosphorylation and the HCV RNA levels in the infected cells (Fig 3E–3H), the shRNA-mediated CaMKII (γ or δ) knockdown did not affect S235 phosphorylation (Fig 5A and 5B). In fact, shRNA-mediated CaMKII δ knockdown resulted in an inverse correlation with the HCV RNA levels in the infected Huh7.5.1 cells (Fig 5C). Potential explanations to the above discrepancy include promiscuousness of the small chemical-based method that results in global effects leading to reduced S235 phosphorylation and reduced HCV RNA levels (Fig 3E–3H). Compared to the chemical-based method, the shRNA sequence-based gene knockdown is likely to result in CaMKII-specific inhibition. Because CaMKII (γ or δ) knockdown did not affect NS5A S235 phosphorylation (Fig 5A and 5B), CaMKII (γ or δ) is unlikely to be responsible for NS5A S235 phosphorylation in vivo in the Huh7.5.1 cells. Although the in vitro CaMKII kinase assay results are consistent with a role of CaMKII in NS5A S235 phosphorylation (Fig 4B), there is always a possibility that the "purified" CaMKII kinase contains trace of other kinases that gave false positive results.

Given the fact that NS5A S235 can be phosphorylated by CKIα [12] and CaMKII (γ and δ) in vitro (Fig 4B), it is possible that the lack of effects in the CaMKII (γ or δ) single knockdown cells was a result of compensatory phosphorylation by CKIα. We thus compared the effects of CKIα single knockdown with those of CKIα plus CaMKII (γ or δ) double knockdown. Consistent with the previous finding [12], CKIα single knockdown reduced NS5A S235 phosphorylation in the infected Huh7.5.1 cells (Fig 6B and 6C). CKIα plus CaMKII (γ or δ) double did not further reduce NS5A S235 phosphorylation (Fig 7B and 7C), suggesting that CaMKII (γ or δ) is not the kinase directly responsible for NS5A S235 phosphorylation in vivo. One simplest explanation to the above observations is that CaMKII (γ or δ) and NS5A are in different cellular compartments whereas CKIα is in a close proximity of NS5A for NS5A phosphorylation. Thus, CKIα, compared to CaMKII, is the preferred kinase for NS5A S235 phosphorylation in the HCV-infected Huh7.5.1 cells in vivo.

One interesting observation is that CaMKII single knockdown appeared to elevate the HCV RNA levels in the Huh7.5.1 cells (Fig 5C and 5D). The effects are most apparent in the CaMKII δ knockdown cells where the CaMKII δ knockdown efficiency showed inverse relationship with the levels of the HCV RNA levels (Fig 5C). This inverse relationship persisted in the CaMKII plus CKIα double knockdown cells (Fig 7D), despite successful reduction of NS5A S235 phosphorylation upon CKIα knockdown (Fig 7B and 7C). The above observations suggest that CaMKII (γ and δ) probably has a negative role in the HCV life cycle. Additional investigation is needed to warrant the roles of CaMKII. In conclusion, the NS3-5A expressing HEK293T platform can be expanded to a larger scale to screen for kinases responsible for NS5A phosphorylation. CKIα as well as CaMKII (γ and δ) can directly phosphorylate NS5A S235 in vitro. CKIα is a major kinase for NS5A S235 phosphorylation and functions in the HCV-infected Huh7.5.1 cells in vivo.

## Materials and Methods

### Cells and plasmids

The human hepatocellular carcinoma Huh7.5.1 cell line originated in Francis V. Chisari’s laboratory in the Scripps Research Institute was maintained as described previously [26]. The human embryonic kidney HEK293T cell line was cultured in Dulbecco’s Modified Eagle Medium (DMEM, 12100046, ThermoFisher Scientific) plus 10% fetal bovine serum (FBS) in a 37°C incubator containing 95% air and 5% CO_2_. The wild type HCV full length viral construct without (J6/JFH-1 chimera, genotype 2a) or with the Renilla luciferase reporter (5’C19Rluc2AUbi, J6/JFH-1 chimera) were gifts from Charles M. Rice in the Rockefeller University [27, 28]. The miR-122 plasmid was obtained from Yoshiharu Matsuura in the Osaka University [19]. The firefly reporter construct (CAT‐IRES^EMCV^‐FLuc) was a gift from Penelope Mavromara [29]. The pcDNA3.1(+)-NS3-NS5A expression construct was made in the Biomedical Resource Core of the First Core Facility in the National Taiwan University College of Medicine. Briefly, an NS3-NS5A fragment was amplified via polymerase chain reaction (PCR) from the 5’C19Rluc2AUbi construct and inserted into the pSTBlue-1 vector (70199, EMD Millipore) via TA cloning. The NS3-5A fragment was then excised via HindIII/XbaI double digestion and inserted into the pcDNA3.1(+) vector (V79020, Invitrogen) after the cytomegalovirus promoter.

### In vitro transcription

The full length HCV constructs, J6/JFH1 or J6/JFH1(5’C19Rluc2AUbi), were linearized with an overnight Xbal digestion prior to phenol-chloroform extraction. The linearized construct was then used as a template to produce the viral RNA using the MAGAscript T7 in vitro transcription kit (AM1334, Ambion). The viral RNA was then purified with phenol-chloroform extraction and isopropanol precipitation. The quality and quantity of the viral RNA were assessed with 0.8% agarose gel electrophoresis and the NanoDrop spectrophotometer.

### Reporter virus assay

The Huh 7.5.1 cells (or HEK293T cells, 3x10^6^) were seeded in a 6-cm petri dish and cultured for 24 hours. Before transfection, the medium was replaced with the Opti-MEM® I Reduced Serum Medium (31985070, ThermoFisher Scientific). The cells were then transfected with the firefly reporter construct (CAT-IRES^EMCV^-FLuc, 150 ng) as an internal control using the Lipofectamine® 2000 transfection reagent (11668–019, ThermoFisher Scientific). For the miR-122 expressing HEK293T cells, the HEK293T cells were co-transfected with the CAT-IRES^EMCV^-FLuc construct and the miR122 construct (8 μg). Twenty-four hours later, the transfected cells were reseeded in a 6-well plate at a density of 1x10^6^ cells per well and then cultured for 24 hours. The cells were then transfected with the J6/JFH1(5’C19Rluc2AUbi) reporter viral RNA using the DMRIE-C reagent (10459014, ThermoFisher Scientific). Four hours later, the medium was replaced with the DMEM (12100046, ThermoFisher Scientific) containing 10% FBS. Thereafter, the cell lysates were collected 4, 12, 24, 48 and 72 hours via 2 washes with phosphate-buffered saline (PBS, 10mM Na_2_HPO_4_, 1.8mM KH_2_PO_4_, 137 mM NaCl and 2.7mM KCl, pH7.4) and lysis in 500 μl 1X Passive Lysis Buffer (E1941, Promega). The cell lysates were centrifuged at 10,000 xg for 10 minutes at 4°C. Twenty μl of the supernatant were subjected to firefly and Renilla luciferase activity measurements using the Dual-Luciferase® Reporter Assay System (E1960, Promega) and the Orin microplate luminometer (Titertek-Berthold).

### HCV virus production

In vitro transcribed J6/JFH-1 viral RNA was transfected in to the Huh7.5.1 cells for 72 hours. The virus-containing culture medium was then collected every day for a week. The collected medium was centrifuged at 3,000 xg for 30 minutes to remove cell debris before the supernatant was used to concentration the virus with the 100kDa Amicon Ultra-15 centrifugal filter (UFC910008, Millipore). The viral titer was determined with the method of serial dilution (10X) that resulted in infection i.e. NS5A positive as detected with immunofluorescence staining using the 2F6 antibody from BioFront technologies. The virus was stocked at -80˚C before use.

### Immunofluorescence staining

Huh7.5.1 cells (10^4^ cells per well) were seeded in a 96-well plate and infected with HCV (J6/JFH1) for 3 days. The Huh7.5.1 cells were washed with ice cold PBS, fixed with 4% paraformaldehyde in PBS for 20 minutes, treated with Triton X-100 (0.3% in PBS) for 30 minutes, rinsed 3 times with PBS and blocked with PBS containing 1% bovine serum albumin (BSA), 0.05% saponin and 0.2% gelatin for 30 minutes. The cells were then incubated with anti-NS5A primary antibody (2F6), washed and incubated with the Alexa-488 labeled secondary antibody. Cell nuclei were counterstained with DAPI (D9542, Sigma-Aldrich). Immunofluorescence images were taken using a Zeiss Axio Vert.A1 microscope and software.

### Immunoblotting

Huh7.5.1 (or HEK293T) cells were harvested in the IP lysis buffer (8M Urea, 75mM NaCl and 50 mM Tris, pH8.0) containing protease inhibitor cocktail (539134, Merck Millipore) and phosphatase inhibitor cocktail (524625, Merck Millipore) before the protein concentrations were quantified with the BCA Protein Assay Kit (23225, ThermoFisher Scientific). Protein samples (20 μg) were separated via 7.5% sodium dodecyl sulfate polyacrylamide gel electrophoresis and transferred to a nitrocellulose membrane (162–0112, Bio-Rad). The membrane was blocked with 0.1% BSA dissolved in tris-buffered saline (20mM Tris, 150mM NaCl, pH7.4 plus 0.1% Tween-20) before protein detection with antibodies. The primary antibodies were NS5A (7B5, BioFront technologies) and β-actin (A5316, Sigma-Aldrich). In some cases, Coomassie blue staining was used to control for total protein loadings. The NS5A S235 phosphorylation-specific antibody was characterized previously [12]. The infrared dye-conjugated secondary antibodies (IRDye 680 and IRDye 800) were obtained from Li-Cor. Protein abundance was quantified with the Li-Cor Odyssey fluorescence imaging system.

### NS3-5A expressing HEK293T cell-based protein kinase screening

To establish a cell-based kinase screening system, the HEK293T cells (10^6^ cells per well) were seeded in a 6-well plate and transfected with the pcDNA3.1(+)-NS3-5A expression construct (3 μg) using the Lipofectamine® 2000 reagent. Twenty-four hours later, the cells were exposed to calmodulin inhibitor W7 (0369, Tocris Bioscience), calmodulin-dependent kinase inhibitor KN93 (13319, Cayman), casein kinase I inhibitor D4476 (D1944, Sigma) or polo-like kinase I inhibitor BI2536 (LT-P0424, Lumtec, Taiwan) for 48 hours before the cell protein was harvested for immunoblotting.

### MTT cell viability assay

HEK293T cells (or HCV-infected Huh7.5.1 cells, 7.5x10^3^ per well) were seeded in a 96-well plate. Twenty-four hours later, the cells were exposed to the kinase inhibitors before the cell viability assay using the MTT (3-(4,5-Dimethyl-2-thiazolyl)-2,5-diphenyl-2H-tetrazolium bromide, M2128, Sigma) reagent, which was converted to formazan by the NADPH-dependent cellular oxidoreductase in viable cells. Briefly, the medium containing the drugs was removed from the well and 50 μl of MTT reagent were added to the wells. The plate was incubated for 1 hour before 100 μl dimethyl sulfoxide (D8418, Sigma) were added to the wells. After a gently shaking, the plate was subjected to formazan measurements at 570nm wavelength in a microplate spectrophotometer (PowerWave XS, BioTek Instruments, Inc.). For the shRNA knockdown cells, the cell viability was assessed on the 6^th^ day after the knockdown.

### Huh7.5.1 cell-based kinase inhibitor assay

Huh7.5.1 cells (2x10^6^) were seeded in a 10-cm dish. After 24 hours, the Huh7.5.1 were infected with HCV (J6/JFH1) for 72 hours. The infected cells were detached from the petri dishes with trypsin digestion and reseeded in a 6 well plate at a density of 5x10^5^ cells per well. Twenty-four hours later, W7 or KN93 was used to treat the cells for 24 hours before the cell protein was harvested for immunoblotting. In a separate experiment, RNA was extracted for quantitative RT-PCR analysis.

### Quantitative and non-quantitative RT-PCR analysis

The cells were dissolved in the TRIzol® reagent (15596018, ThermoFisher Scientific) before the total RNA were extracted with the Direct-zol RNA MiniPrep (R2052, ZYMO Research). To synthesize cDNA, the total RNA was subjected to reverse transcription using the SuperScript III reverse transcriptase (18080044, ThermoFisher Scientific) and random hexamer primers. Gene-specific PCR products were generated with primers specific to HCV RNA, CKIα, CaMKII γ and CaMKII δ (Table 1). Primers were synthesized by Integrated Device Technology. All samples were analyzed in triplicates. The glyceraldehyde 3-phosphate dehydrogenase (GAPDH) mRNA levels were measured as internal controls. Reactions without template were included as negative controls. To detect CaMKII isoform mRNA expression in the Huh7.5.1 cells, non-quantitation RT-PCR was performed using primers that span two neighboring exons or that are within the same exon (Table 2).

**Table 1:** Primers used for quantitative RT-PCR analysis.

Primer	Sequence 5’ – 3’
HCV 5’ UTR F	TCTGCGGAACCGGTGAGTA
HCV 5’ UTR R	TCAGGCAGTACCACAAGGC
CKIα F	CATCTATTTGGCGATCAACATCA
CKIα R	GCCTGGCCTTCTGAGATTCTA
CaMKIIγ F	GTGGAGTGTTTGCGCAAGTT
CaMKIIγ R	TGACACCGCCATCCGACT
CaMKIIδ F	GGAATTTCTCAGCAGCCAAG
CaMKIIδ R	GCTTTCGTGCTTTCACATCT
GAPDH F	GCCCTCAACGACCACTTTGT
GAPDH R	TGGTGGTCCAGGGGTCTTAC

CaMKII, calmodulin-dependent kinase II; CKIα, casein kinase I α; F, forward primer; GAPDH, glyceraldehyde 3-phosphate dehydrogenase; R, reverse primer; UTR, untranslated region.

**Table 2:** RT-PCR primers used for CaMKII isoform analysis.

Primer	Type	Sequence 5’ – 3’
CaMKIIα F	exon-exon junction	TGGAATCCTCAGAGAGCACC
CaMKIIα R	exon-exon junction	CGCACATCTTCGTGTAGGACT
CaMKIIβ F	exon-exon junction	AGTCAAGCCCCAGACGAATA
CaMKIIβ R	exon-exon junction	GCACTGTCAGAAGACTCCTT
CaMKIIγ F	exon-exon junction	GTGGAGTGTTTGCGCAAGTT
CaMKIIγ R	exon-exon junction	TGACACCGCCATCCGACT
CaMKIIδ F	exon-exon junction	GGAATTTCTCAGCAGCCAAG
CaMKIIδ R	exon-exon junction	GCTTTCGTGCTTTCACATCT
CaMKIIα F	exon primer	ATCGCCTACATCCGCATC
CaMKIIα R	exon primer	CCAGATCTGTGGAAGTGGAC
CaMKIIβ F	exon primer	GTTGTTCCTGCTTTCCCTTC
CaMKIIβ R	exon primer	TTTCAATACTCCAGGGGGTG

The exon-exon primers span two neighboring exons whereas the exon primers are within the same exon of a gene. F, forward primer; R, reverse primer.

### In vitro kinase assay

Active calmodulin-dependent kinase II γ and δ were purchased from SignalChem (C13-10G-05 and C14-10BG-05). Two NH_2_-terminally biotin-labeled peptides mapped to amino acids 216–243 of NS5A were synthesized by GeneTex Corp. The primary sequences for the NS5A-101 and NS5A-111 peptides are respectively biotin-RRLARGsPPSEASSSVSQLSAPsLRATC and biotin-RRLARGsPPSEASSSVSQLsAPsLRATC, where the lower case s indicates phosphorylated serine. Following the manufacturer's instruction, the active kinase was mixed with the NS5A-101 peptide on ice. The ATP solution was then added to the mixture and incubated at 30°C for 15 min prior to detection of S235 phosphorylation using dot blotting. IRDye 800 conjugated streptavidin was used to detect the biotin-labeled peptide for loading control purpose.

### Production of lentivirus carrying a gene-specific shRNA sequence

Gene-specific small hairpin RNA (shRNA) carrying plasmids were purchased from the RNAi core facility in the Academia Sinica, Taiwan (Table 3). To generate lentivirus carrying a gene-specific shRNA sequence, the HEK293T cells were cultured in a 6-cm petri dish with 3 ml DMEM medium containing 1% BSA for 30 minutes before they were transfected with 3 plasmids: the plasmid carrying gene-specific shRNA sequence (4 μg), the VSVG plasmid (0.4 μg) and the PCMV plasmid (3.6 μg) dissolved in 250 μl Opti-MEM® I Reduced Serum Medium. To each petri dish, 12 μl of the T-pro NTRII reagent (JT97-N002, T-PRO Biotechnology, Taiwan) were added to enhance transfection efficiency. Two days after the transfection, the medium containing lentivirus was harvested and centrifuged 1,100 xg for 5 minutes before the supernatant was stored in a -80°C freezer.

**Table 3:** Plasmids used for shRNA-mediated gene knockdown.

shRNA clone (ID)	Sequence	Target
shCon (TRCN0000072246)	CAAATCACAGAATCGTCGTAT	no target in the Huh7.5.1 cells
shCKIα(TRCN0000342507)	GCAGAATTTGCGATGTACTTA	coding region
shCaMKIIγ (1) (TRCN0000000476)	CGTTGCTGTACTGTCTTGTTT	3’ UTR
shCaMKIIγ (2) (TRCN0000000478)	AGAACAGCAAGCCTATCCATA	coding region
shCaMKIIδ (1) (TRCN0000195619)	CCATGGATCTGTCAACGTTCT	coding region
shCaMKIIδ (2) (TRCN0000000474)	CAGATGGAGTAAAGGAGTCAA	coding region
shCaMKIIδ (3) (TRCN0000000475)	TGTGGTGTCATTCTCTATATT	coding region

CaMKII, calmodulin-dependent kinase II; CKIα, casein kinase I α; shCon, non-targeting control shRNA plasmid.
